# A Nomogram Combined Radiomics and Kinetic Curve Pattern as Imaging Biomarker for Detecting Metastatic Axillary Lymph Node in Invasive Breast Cancer

**DOI:** 10.3389/fonc.2020.01463

**Published:** 2020-08-28

**Authors:** Yan-na Shan, Wen Xu, Rong Wang, Wei Wang, Pei-pei Pang, Qi-jun Shen

**Affiliations:** ^1^Department of Radiology, Affiliated Hangzhou First People's Hospital, Zhejiang University School of Medicine, Hangzhou, China; ^2^Department of Ultrasound, Affiliated Hangzhou First People's Hospital, Zhejiang University School of Medicine, Hangzhou, China; ^3^GE Healthcare (China), Hangzhou, China

**Keywords:** axillary lymph node, breast cancer, algorithm, magnetic resonance imaging, radiomics

## Abstract

**Objective:** To construct and validate a nomogram model integrating the magnetic resonance imaging (MRI) radiomic features and the kinetic curve pattern for detecting metastatic axillary lymph node (ALN) in invasive breast cancer preoperatively. Materials and Methods: A total of 145 ALNs from two institutions were classified into negative and positive groups according to the pathologic or surgical results. One hundred one ALNs from institution I were taken as the training cohort, and the other 44 ALNs from institution II were taken as the external validation cohort. The kinetic curve was computed using dynamic contrast-enhanced MRI software. The preprocessed images were used for radiomic feature extraction. The LASSO regression was applied to identify optimal radiomic features and construct the Radscore. A nomogram model was constructed combining the Radscore and the kinetic curve pattern. The discriminative performance was evaluated by receiver operating characteristic analysis and calibration curve.

**Results:** Five optimal features were ultimately selected and contributed to the Radscore construction. The kinetic curve pattern was significantly different between negative and positive lymph nodes. The nomogram model showed a better performance in both training cohort [area under the curve (AUC) = 0.91, 95% CI = 0.83–0.96] and external validation cohort (AUC = 0.86, 95% CI = 0.72–0.94); the calibration curve indicated a better accuracy of the nomogram model for detecting metastatic ALN than either Radscore or kinetic curve pattern alone.

**Conclusion:** A nomogram model integrated the Radscore and the kinetic curve pattern could serve as a biomarker for detecting metastatic ALN in patients with invasive breast cancer.

## Introduction

Axillary lymph node (ALN) status in patients with invasive breast cancer is a vital information for guiding therapy and evaluating prognosis (1–3). Axillary lymph node dissection (ALND) would be performed for defining ALNs status if the sentinel lymph nodes were positive for metastasis. However, ALND is associated with significant complications (4, 5). But based on the result of a multicenter randomized trial from the American College of Surgeons Oncology Group (ACOSOG) Z0011 (6), 2014 American Society of Clinical Oncology guideline recommended that ALND may not be necessary in T1 or T2 stage breast cancer patients with one or two positive sentinel lymph nodes who are being treated with breast-conserving therapy and adjuvant systemic therapy (7). In this context, it is debatable how aggressively radiologists should perform percutaneous sampling of axillary nodes to identify axillary metastasis. Cody and Houssami (8) reported that preoperative axillary sampling should be reserved for patients with more than 2 abnormal lymph nodes on imaging, and ALND should be performed directly during mastectomy if the results were positive. All remaining patients should undergo sentinel lymph node biopsy (SLNB) in hopes of meeting Z0011 trial eligibility criteria to avoid ALND. Therefore, accurate, non-invasive detection approach may be imperative to distinguish between lymph nodes that were positive for metastasis (“positive” nodes) and those that were negative for metastasis (“negative” nodes) and reducing unnecessary percutaneous sampling of ALNs, or SLNB, or ALND at a certain extent in the post-ACOSOG Z0011 era.

Magnetic resonance imaging (MRI) can visualize morphological and contrast-enhanced characteristics of metastatic lymph nodes. Some qualitative characteristics may cause inevitable inconsistency due to subjectivity (9–12). The threshold value of apparent diffusion coefficient showed inconsistent results in distinguishing between negative nodes from positive nodes due to the low signal-to-noise ratio and image distortion of diffusion-weighted imaging (13, 14). Some quantitative contrast-enhanced parameters (initial enhancement, peak enhancement, and delayed enhancement) represent the enhancement characteristics of the single time phase. The kinetic curve pattern based on dynamic contrast-enhanced MRI (DCE-MRI), which shows the trend of signal intensity in the contrast-enhanced process, is commonly used in clinical settings (15). It had been repeatedly demonstrated to be significantly different between negative and positive ALNs in previous studies (16–18). The kinetic curve was calculated by drawing region of interest (ROI) of focal area. However, large amounts of quantitative imaging information representing underlying histologic characteristics could not be acquired by visual inspection.

Radiomic analysis links quantitative imaging features to clinical findings by using machine-learning and statistics-analysis methods (19–22). The quantitative imaging analysis is expected to identify the imaging features that correlate to the pathophysiology of lesions more objectively (23–26). At present, radiomics is mainly used for the single-phase imaging analysis, which cannot reflect the kinetic characteristics of the lesion (27–29). In this study, we hypothesized that radiomic features and kinetic curve pattern could identify the association between MRI characteristics and the pathophysiology of ALNs and thus effectively and precisely detected potential metastatic ALNs in invasive breast tumors. Accordingly, the aim of the study was to construct a nomogram model based on the integration of quantitative radiomic parameters and the kinetic curve pattern. External validation was then performed to assess the preoperative prediction efficiency of the proposed model, which might contribute to metastatic ALN detection in the individualized precision treatment for invasive breast tumors.

## Materials and Methods

**Figure 1:**
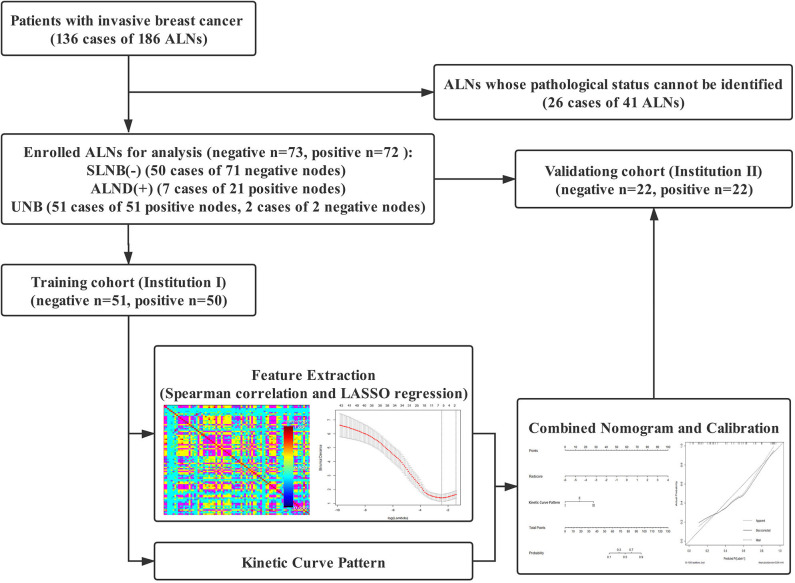
Workflow of the study.

This retrospective study was approved by the medical ethics committee of our institution and was conducted in accordance with relevant guidelines. Informed consent was waived. The workflow of the analysis is summarized in Figure 1.

### Study Population and Surgical Strategy

Patients with suspected breast cancer who underwent DCE-MRI of the breast between January 2018 and December 2019 were retrospectively collected in two institutions. The final diagnosis of invasive breast cancer was based on pathological analysis.

On the affected side, the ALN inclusion criteria for the study were as follows: (1) suspicious node with cortical irregularity (30); (2) the shortest dimension was no <5 mm (10, 30); (3) MRI scans available for qualitative and radiomic analysis; (4) no previous chemotherapy or radiation therapy; and (5) complete medical records including pathological diagnosis and treatment.

The standard of determining negative or positive lymph nodes was established in three ways: (1) all ALNs of the patients with negative SLNB were considered negative (31); (2) the pathological results were confirmed by ultrasound-guided needle biopsy. The suspicious lymph node in the MRI scan was located in the ultrasound image by MRI virtual navigation or by a radiologist and sonographer comparing MRI scans with ultrasonic image together; (3) when multiple lymph nodes (*n* > 8) were proven positive for metastasis at ALND, highly suspicious lymph nodes on MRI (up to three lymph nodes per patient) were presumed positive for metastasis (32).

Exclusion criteria were as follows: (1) the pathological status of lymph node cannot be identified in any of these three ways above; and (2) the tissue for biopsy was damaged and failed to provide meaningful pathological results.

### Imaging Protocol

Dynamic contrast-enhanced MRIs were performed at 3.0-T systems in both institutions (institution I: MAGNETOM Verio, Siemens AG, Germany; institution II: MAGNETOM Skyra, Siemens AG, Germany). Both institutions applied the same imaging protocols. The patient was in the prone position, with both breasts naturally and closely fitted in the breast coil. T1-weighted three-dimensional fluid-attenuated inversion recovery was applied for dynamic contrast enhancement (repetition time/echo time, 4.51/1.61; flip angle, 10°; slice thickness, 1.0 mm; section gap, 0.20 mm; field of view, 320 × 320; image matrix, 420 × 420). Contrast material was injected into the elbow vein [0.1 mmol/kg of gadodiamide (Omniscan, GE Healthcare)] and followed by a 20-mL saline flush at a rate of 2.0 mL/s. After the first precontrast scan, five consecutive postcontrast phases were obtained starting at 25-s delay after contrast injection. Each phase took 59 s.

### Image Processing

As compared with images of T1-weighted, T2-weighted, and diffusion-weighted, contrast-enhancement images had relatively small image noise and distortion, which can more clearly visualize the delineation of ALNs (11). The first postcontrast phase images were chosen for feature extraction because the average peak enhancement at the early postcontrast stage was significantly different between negative and positive ALNs (18, 33), and the distribution of the contrast agent in lesions was more homogeneous in this phase (34). ITK-SNAP software (version 3.6, http://www.itksnap.org) was utilized to segment volumes of interest (VOIs) of ALNs on contrast-enhanced images. Before delineation, gray-level standardization was applied to reduce the gray-level differences caused by the imaging procedure. Each layer contour of VOIs was delineated along the inner margin of the lymph node to avoid the false heterogeneity caused by the unclear edge. The VOI contours were superimposed to improve the consistency of node segmentation. All pixels' gray scales inside the VOIs were extracted for analysis.

The kinetic curve was computed using DCE-MRI software (mean curve: Siemens Healthcare, Germany). A circular ROI of 20 mm^2^ was placed at the lymph node with maximal enhancement determined on the first postcontrast images. The areas without enhancement in the lymph node, which were hypointensity both in precontrast phase and subtraction images, were excluded as necrosis. The kinetic curve pattern was defined according to changes in pixel values, between the second contrast-enhanced and delayed contrast-enhanced series, as follows: persistent type (type I) indicated an increased pixel signal intensity >10% from the second postcontrast series; washout type (type III) indicated decreased pixel signal intensity at the last postcontrast series >10% from the second postcontrast series; plateau type (type II) indicated pixel signal intensity change in neither direction by more than 10%.

Both image segmentation and kinetic curve pattern were evaluated by two breast radiologists (a chief and a resident with 15 and 3 years of experience, respectively) who were blind to the pathology of lymph nodes independently.

### Feature Extraction, Selection, and Correlation

A total of 396 radiomic features were extracted in each VOI using the Artificial Intelligence Kit version 3.0.1.A (Life Sciences, GE Healthcare, US), including six categories of parameters: morphology, histogram, texture parameters, gray-level co-occurrence matrix parameters, gray-level run-length matrix parameters, and gray-level zone size matrix parameters. Details of the procedures for extraction of radiomic features are described in Supplementary Figures 1, 2.

The analysis of variance and Mann–Whitney *U* test (ANOVA-MW) were carried out for selecting significant features that were highly correlated. Spearman correlation test with correlation coefficient more than 0.90 was applied to remove the redundancy; radiomic features were further optimally elected. In the final step, the least absolute shrinkage and selection operator (LASSO) regression method was applied to identify the most nonredundant and robust features from the training cohort in order to improve the class separability and optimize the representation of lesion heterogeneity. The complexity of LASSO regression was controlled by a tuning parameter lambda (λ) with the rule that as the value of λ increases, the penalty for each variable coefficient also increases, and the relevant features with nonzero coefficients were selected and contributed to the final LASSO regression (35). Meanwhile, the best value of λ found by 10-fold cross-validation with a maximum area under the curve (AUC) was used for constructing the regression model. Radscore, which was defined by corresponding nonzero coefficients of features selected by LASSO, was created by a linear combination of selected features weighted by their coefficients. Respective Radscore was calculated for each lymph node.

### Nomogram Building, Calibration, and External Validation

Both the Radscore and the kinetic curve pattern were integrated by a multivariate logistic regression analysis in the training cohort. Based on this, a nomogram was constructed for detecting metastatic ALNs. The receiver operating characteristic (ROC) analysis was applied to evaluate the discrimination performance of the model. Along with the Hosmer–Lemeshow test measuring for goodness of fit of the nomogram, the classification accuracy was assessed via calibration curves. The degree of overlap between the calibration curve and the diagonal in the graph reflects the accuracy of the nomogram model. The constructed nomogram model was validated on external validation cohort using the same process of capability assessment with the ROC analysis and calibration curve.

### Statistical Analysis

Statistical analysis was conducted by R software (version 3.3.2) and MedCalc (version 19.1). Variables of a normal distribution were shown as mean ± SD, and variables of a skew distribution were shown as median (quartile). Statistical group comparisons of data were analyzed by Wilcoxon using rank-sum (continuous variables) and χ^2^ (categorical/dichotomous variables) tests. Intraclass correlation coefficient (ICC) was analyzed for estimating reliability of interobserver agreements including kinetic curve pattern identification and radiomic analysis, which was defined as good consistency between 0.75 and 1; fair consistency, between 0.4 and 0.75; and poor, <0.4. The correlation and collinearity of radiomic features were evaluated using VIF function. The Pearson correlation analysis was performed to evaluate the correlation between kinetic curve pattern and Radscore; the pairwise Pearson correlation coefficients were calculated. The kinetic curve pattern, Radscore, and nomogram model were, respectively, subjected to ROC analysis, by using AUC, sensitivity, specificity, and accuracy to evaluate the stratification efficacy. The comparison of ROC curves was performed by Delong test. The level of statistical significance was set at a two-sided *p* < 0.05.

## Results

### Patients Characteristics

A total of 145 ALNs from 102 patients (age range, 27–83 years; mean age, 52.45 ± 11.78 years) with invasive breast cancer were selected in the final cohort. Seventy-six cases of 101 ALNs (51 negative nodes, 50 positive nodes) from institution I were taken as the training cohort, and the other 34 cases of 44 ALNs (22 negative nodes, 22 positive nodes) from institution II were taken for external validation. Table 1 shows the kinetic curve pattern of ALNs in two cohorts. It was significantly different in both cohorts (*p* < 0.001).

**Table 1:** Kinetic curve pattern of ALNs in training cohort and validation cohort.

**Kinetic curve pattern**	**Training cohort**		***p*-value**	**Validation cohort**		***p*-value**
	**Negative (*n* = 51)**	**Positive (*n* = 50)**		**Negative (*n* = 22)**	**Positive (*n* = 22)**	
Type I	19	3	<0.01	10	2	0.01
Type II	24	22		8	9	
Type III	8	25		4	11	

### Reproducibility Analysis

For identifying the kinetic curve pattern, the ICC was 0.98 between two breast radiologists, indicating satisfactory consistency. Based on the result of reproducibility analysis by two radiologists, 363 of 396 (91.7%) radiomic features had good consistency (ICC ≥ 0.75). The numbers of features with fair consistency (0.75 > ICC ≥ 0.4) and poor consistency (ICC <0.4) were 19 (4.8%) and 14 (3.5%), respectively. Table 2 shows the ICC value of significant features.

**Table 2:** Reproducibility analysis of significant features.

**Significant feature**	**Feature class**	**ICC (95% CI)**
Kinetic curve pattern		0.98 (0.97–0.99)
Uniformity	Histogram parameters	0.93 (0.82–0.97)
Correlation_a135_o1	Texture parameters	0.87 (0.78–0.98)
Inertia_a90_o4	Texture parameters	0.90 (0.85–0.96)
CP_all_o1_SD	Texture parameters	0.79 (0.69–0.82)
SVR	Form factor parameters	0.96 (0.87–0.99)

*Correlation_a135_o1, Correlation_angle135_offset1; Inertia_a90_o4, Inertia_angle90_offset4; CP_all_o1_SD, ClusterProminence_AllDirection_offset1_SD; SVR, surface volume ratio*.

### Radscore Model Building, Kinetic Curve Pattern Analysis, Correlation, and Validation

After dimensionality reduction, which included ANOVA and MW (251 features), Spearman correlation test (96 features), and the LASSO algorithm with the optimal regulation weight λ [log(λmin) = −2.46], five radiomic features with nonzero coefficients were finally selected by 10-fold cross validation to ensure robustness and preventing overfitting. To demonstrate the effectiveness of radiomic features model at the individual scale, the quantitative values of radiomic features for each lymph node the classification of negative and positive groups in training cohort are shown in Table 3, which included uniformity, Correlation_angle135_offset1 (Correlation_a135_o1), Inertia_angle90_offset4 (Inertia_a90_o4),ClusterProminence_AllDirection_offset1_SD (CP_all_o1_SD), and surface volume ratio. A Radscore model was further constructed based on five features with respective nonzero coefficients selected through LASSO regression method. There was no collinearity between the five features after being verified by VIF function. The complete details are shown in Supplementary Figure 3.

$$\begin{array}{l} \text{Radscore}=-0.028-0.692\times \text{Uniformity}+0.188\\ \;\times \text{Correlation\_all\_}o1-0.267\times \text{Inertia\_a}90\text{\_o}1\\ \;+0.522\times CP\text{\_all\_}o1-0.954\times \text{SVR} \end{array}$$

**Table 3:** Univariate analysis of significant features in the training cohort.

**Significant features**	**Negative (*n* = 51)**	**Positive (*n* = 50)**	***p***
Uniformity	0.38 ± 0.99	−0.39 ± 0.86	<0.01
Correlation_all_o1	−0.43 ± 0.70	0.44 ± 1.07	<0.01
Inertia_a90_o4	−0.41 ± 1.03	−0.42 ± 0.78	<0.01
CP_all_o1	−0.36 ± 1.04	−0.36 ± 0.62	<0.01
SVR	0.46 ± 0.83	−0.46 ± 0.95	<0.01

*Correlation_a135_o1, Correlation_angle135_offset1; Inertia_a90_o4, Inertia_angle90_offset4; CP_all_o1_SD, ClusterProminence_AllDirection_offset1_SD; SVR, surface volume ratio*.

**Figure 2:**
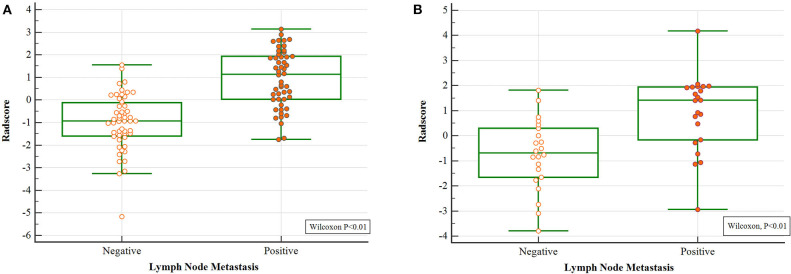
Wilcoxon analysis of Radscore for detecting metastatic ALN in the **(A)** training cohort and **(B)** validation cohort (*p* < 0.05).

Differences of the Radscore value between the negative and positive ALNs in training and validation cohort were statistically significant (Figure 2).

**Figure 3:**
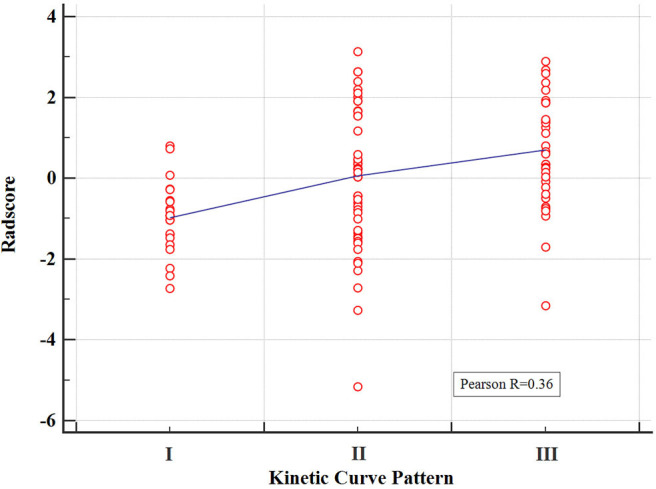
Correlation between the kinetic curve pattern and the Radscore based on Pearson correlation analysis. The mean absolute correlation coefficient was 0.36.

The pairwise Pearson correlation analysis revealed that the Radscore was moderately correlated to kinetic curve pattern (Figure 3).

**Figure 4:**
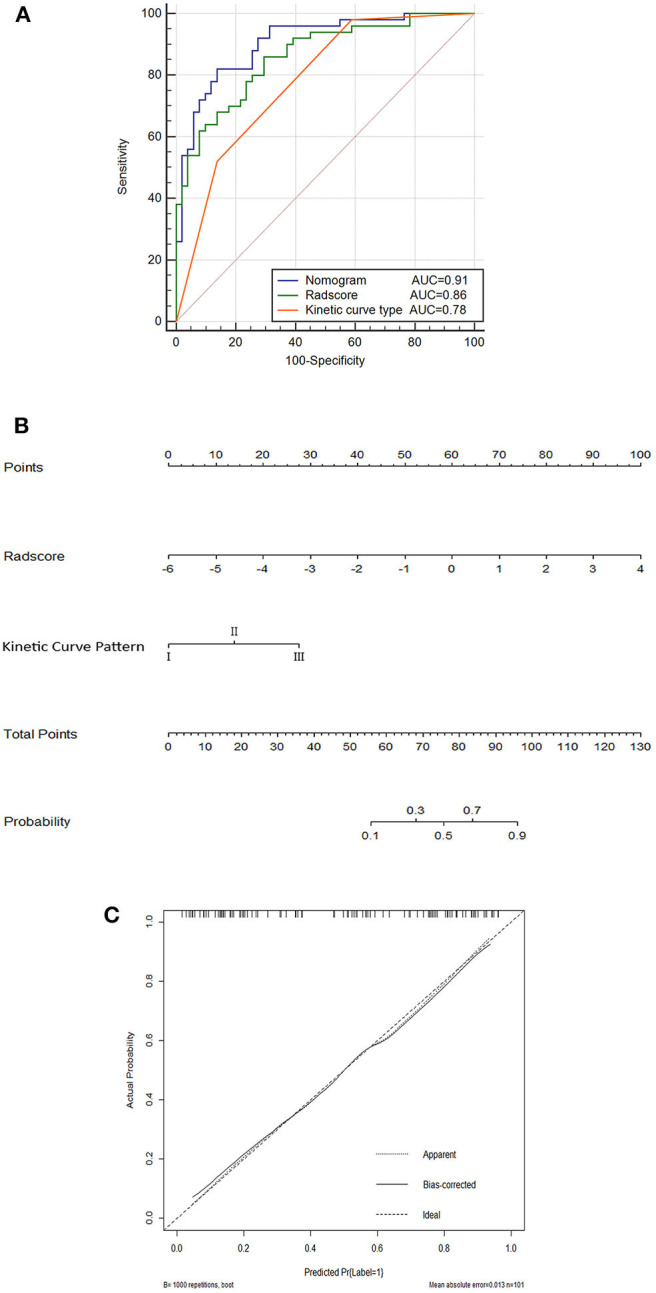
Nomogram **(A)**; ROC curves for the kinetic curve pattern, the Radscore, and the nomogram model **(B)**; and corresponding calibration curves based on the nomogram model **(C)** in the training cohort.

Further validation was carried out through ROC analysis for the detection performance of Radscore, kinetic curve pattern, and the nomogram model in the training cohort. A favorable classification capability was observed with a good AUC in the training cohort (Figure 4A).

### Nomogram Building

The nomogram based on both Radscore and kinetic curve pattern was constructed to visualize the results of multivariable logistic regression analysis for detecting ALN metastasis (Figure 4B).

$$\begin{array}{l} \text{Nomogram}=-2.250+0.932\times \text{Radscore}+1.090\\ \;\times \text{kinetic curve pattern} \end{array}$$

The total points accumulated by the various variables correspond to the metastasis probability for a ALN (36). The complete details are shown in Figure 4B.

The calibration curves and the Hosmer–Lemeshow test of nomogram in the training cohort demonstrated a high accuracy of the model in the stratification capability (Figure 4C). Compared to the Radscore and the kinetic curve pattern alone, the nomogram model yielded a better performance in detecting ALN metastasis including an increased AUC and higher sensitivity, specificity, and accuracy in the training cohort (Table 4). Specifically, the nomogram showed a significant improvement than the Radscore and the kinetic curve pattern along in the Delong test (*p* < 0.05). The details are shown in Supplementary Table 1.

**Table 4:** Performance of the kinetic curve type, Radscore, and the nomogram models.

**Model**	**Training cohort**				**Validation cohort**			
	**AUC (95% CI)**	**SEN**	**SPEC**	**ACC**	**AUC (95% CI)**	**SEN**	**SPEC**	**ACC**
Kinetic curve pattern	0.78 (0.69–0.85)	0.98	0.42	0.69	0.74 (0.58–0.86)	0.91	0.45	0.68
Radscore	0.86 (0.78–0.92)	0.86	0.71	0.78	0.81 (0.67–0.91)	0.68	0.91	0.79
Nomogram	0.91 (0.83–0.96)	0.82	0.86	0.84	0.86 (0.72–0.94)	0.73	0.91	0.82

*AUC, area under the ROC curve; SEN, sensitivity; SPEC, specificity; ACC, accuracy*.

### Validation on External Cohort

**Figure 5:**
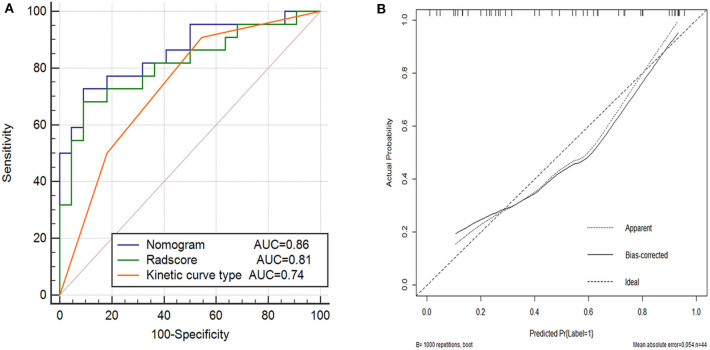
Performance of the kinetic curve pattern, the Radscore and the nomogram model on external validation cohort. **(A)** Receiver operating characteristic curve for the three models with AUCs of 0.74, 0.81, and 0.86, respectively. **(B)** Calibration curve of the nomogram model in the validation cohort.

The performance of the nomogram model was validated using the external dataset collected from the other institution. The nomogram yielded a favorable AUC value in the validation cohort (Figure 5A). The calibration curves and the Hosmer–Lemeshow test of the proposed nomogram model based on the validation cohort suggested a favorable stratification performance (Figure 5B). The nomogram showed a significant improvement than kinetic curve pattern (*p* < 0*.05*), but no significant difference vs. Radscore in the Delong Test (*p* = 0.36). The details are shown in Supplementary Table 2.

## Discussion

In this study, we established and validated a nomogram model to detect metastatic ALNs in patients with invasive breast cancer, which incorporated the kinetic curve pattern and five robust radiomic features extracted from contrast-enhanced MRI. The nomogram model achieved a better performance in both training cohort and external validation cohort with a larger AUC value than the radiomic model or the kinetic curve pattern alone, suggesting the reliability of the improved model in detecting metastatic ALNs.

The kinetic curve pattern is a dynamic feature reflecting the changes of capillary permeability and hemodynamics in the lesion. The infiltration of tumor cells into lymph node promotes the abnormal angiogenesis, damages the normal vessel wall, and forms arteriovenous fistula. These accelerate the efflux of the contrast agent in the delayed phase, and type III kinetic curve is suggestive of positive node. Compared with positive node, the contrast agent circulates slowly in negative node because of the normal capillary network and lower capillary permeability. Type I kinetic curve is suggestive of negative node (37). In this study, the kinetic curve pattern showed significant differences between negative and positive nodes with high sensitivity, but the specificity was relatively low, which leads to the weak diagnostic efficiency that in agreement with previous studies (16, 18, 33, 38). We speculated that the necrosis excluded from the ROI of kinetic curve is a prominent feature for positive node. The kinetic curve pattern focuses on the trend of signal intensity in the contrast-enhanced process, but fails to reflect the heterogeneity of the whole lymph node, which contains large amounts of quantitative features that cannot be observed with the naked eyes.

As previously reported, several imaging findings have been observed for assessing ALN metastasis. Irregularly increased cortical thickness, an absence of the hilum, reduced ratios of the longest to the shortest dimension, and heterogeneous enhancement were suggestive of metastatic lymph node using univariate analysis (9, 10, 33). However, the imaging findings were still a subjective judgment that involved interobserver disagreement, such as the interpretation of morphological and contrast-enhanced characteristics, which had different scoring system in different studies (9–12, 39, 40). Radiomic features, as objective quantitative imaging biomarkers, reflected the heterogeneity of the whole lymph node objectively and could be imperative complementation for detecting the metastatic ALNs. In this study, five radiomic features of 396 radiomic features were selected including uniformity, Correlation_angle135_offset1 (Correlation_a135_o1), Inertia_angle90_offset4 (Inertia_a90_o4), Cluster Prominence_AllDirection_offset1_SD (CP_all_o1_SD), and surface volume ratio, suggesting their vital roles in detection. Uniformity reflects the regularity of gray scale image texture. The texture parameters of correlation, inertia, and cluster prominence represent the distribution and relationship of pixel gray scale in the image. The four optimal features mentioned above indicated the complexity and heterogeneity of the positive node in different categories of textures. It verified that the positive node appeared to be more heterogeneity in the layout of histologic internal components than negative node in contrast-enhanced images because of the infiltration of tumor cells, abnormal angiogenesis, and necrosis. Surface volume ratio is a morphological parameter that reflects the shape and roundness of the lesion. Tumor cells proliferated in the lymph node. The irregular increased cortical thickness and the absence of the hilum led to the transformation of positive node from renal to spherical. The surface volume ratio of lymph node reflects the extent of this transformation. The Radscore of positive nodes was higher than that of negative nodes in both two cohorts, which suggested that positive nodes had greater heterogeneity, as evidenced by the uneven distribution of gray scales and unorganized local texture on the MRI scans. The AUCs of Radscore in training and validation cohorts were 0.86 and 0.81, respectively.

To our knowledge, some studies reported radiomics-related methods for detecting metastatic lymph node (41, 42). However, these models were built based on radiomic features of one phase, which did not include radiomic dynamic features. Up to now, most of the image registration methods of DCE sequences were based on pixel gray level, and they were predominantly counted on the general character of image gray level (43). After contrast agent injection, the gray scale of the lesion changes dramatically, which was significantly different from the surrounding tissues, resulting in a certain extent of distortion in the lesion contour. The enhanced lesion was different from the actual size and shape. Some studies had carried out a certain extent of optimization; however, the precondition of these optimal methods is that ROI has a clear boundary, and the internal tissue gray scale is relatively uniform (44). Therefore, image registration has become the key of dynamic radiomics, which is still controversial at present.

Radiomic features represented underlying histologic characteristics that could not be acquired by visual inspection. Meanwhile, the kinetic curve pattern represented the kinetic process of contrast-enhancement, which could not be extracted by radiomic analysis. Owing to the complement of radiomics and the kinetic curve pattern, the nomogram model represented more effective and reliable than the radiomic model or the kinetic curve pattern alone according to the results of the ROC and calibration analysis. The detection performance of the nomogram model was validated using an external cohort, demonstrating a strong confirmation of reproducibility by a satisfactory AUC of 0.86. Because of the detection of ALNs with high possibility of metastasis before surgery, the nomogram incorporates five selected radiomic features, and the kinetic curve pattern could offer a clinically translatable paradigm easy to implement in the clinical setting.

Although the two radiologists who worked on radiomic analysis differed significantly in their years of experience, the contouring results were relatively consistent (ICC > 0.75). The advantage of a fully quantitative radiomic assessment method is that a wealth of experience in imaging diagnosis is not required. Even a junior physician can accurately delineate ALNs on contrast-enhanced images and preliminarily detect the metastatic ALNs.

There were several limitations in current study that still needs to be further investigated. (1) This was a retrospective study with a relatively small dataset in both training cohort and external validation cohort, and further prospective studies are expected to verify the conclusions. (2) In the process of ALN segmentation, it was prone to cause inaccurate delineation when it abuts the blood vessel and muscle, because of the partial volume effect. (3) The kinetic curve reflects the focal dynamic contrast-enhancement characteristic of the lymph nodes. (4) The feature extraction software made the displacement vectors 1, 4, and 7 to describe the relationship between the gray scale of pixels of the texture as default setting. In light of different set point that could possibly influence the quantity and category of radiomic features extraction, a future radiomic analysis based on various displacement vectors is required. (5) The reproducibility of radiomics in MRI scans with different magnetic field intensities or different brands of MR equipment is expected to be verified in future studies.

## Conclusion

Nomogram-integrated radiomic features and the kinetic curve pattern can be a reliable and effective model for detecting metastatic ALNs in patients with invasive breast cancer. The nomogram could serve as a reliable and convenient tool for ALNs management, suggesting great potential for clinical applications.

## Data Availability Statement

The datasets generated for this study are available on request to the corresponding author.

## Ethics Statement

The studies involving human participants were reviewed and approved by Ethics committee of the Affiliated Hangzhou First People's Hospital, Zhejiang University School of Medicine. Written informed consent for participation was not required for this study in accordance with the national legislation and the institutional requirements.

## Author Contributions

YS contributed to prepare the manuscript and the statistical analysis. QS put forward the concept of the study, designed the study, and reviewed the manuscript. WX, WW, and RW contributed to the data acquisition, analysis, and interpretation. PP carried out the data analysis. All authors contributed to the article and approved the submitted version.

## Conflict of Interest

PP was employed by the company GE Healthcare. The remaining authors declare that the research was conducted in the absence of any commercial or financial relationships that could be construed as a potential conflict of interest.
