# Supplementary material for: A Nomogram Combined Radiomics and Kinetic Curve Pattern as Imaging Biomarker for Detecting Metastatic Axillary Lymph Node in Invasive Breast Cancer
Source: Front Oncol. 2020 Aug 28;10:1463. doi: 10.3389/fonc.2020.01463 (PMC7483545; doi:10.3389/fonc.2020.01463)
Supplement: Supplementary file 1 [file Data_Sheet_1.docx]

***Supplementary Materials***

1. **Feature extraction**

A total of 396 radiomic features were extracted from each ALN in this study. Features pre-processing was conducted in two steps before data dimension reduction. Step 1, outliers and null values were replaced by medial values, and step 2, values standardization was carried out to eliminate the influence of the dimension.

According to *P*＜0.05，ANOVA-MW (The analysis of variance and Mann Whitney U test) were carried out for selecting significant features that were highly correlated (n=251). Spearman correlation test with correlation coefficient more than 0.90 was applied to remove the redundancy, radiomic features (n=95) were further optimally elected (Figure S1). In the final step, the LASSO algorithm with the optimal regulation weight λ= 0.0878 with log (λ) = -2.46, 5 features with non-zero coefficients were finally selected by 10-fold cross validation for ensuring robustness and preventing overfitting (Supplementary Figure 1).


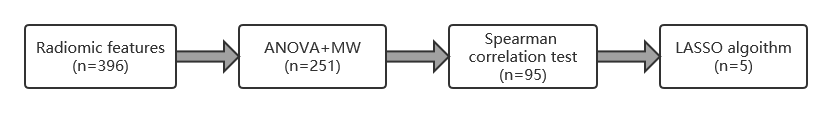


**Supplementary Figure 1.** Workflow of feature extraction.

1. **The least absolute shrinkage and selection operator (LASSO) method**


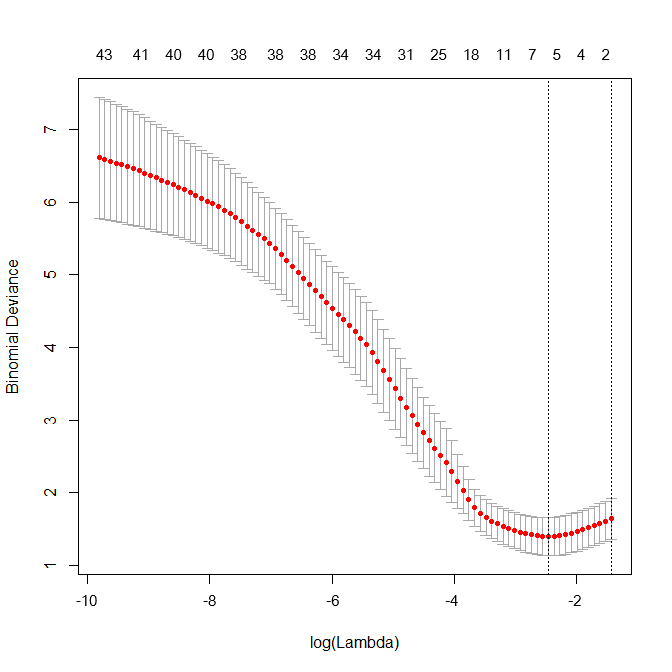

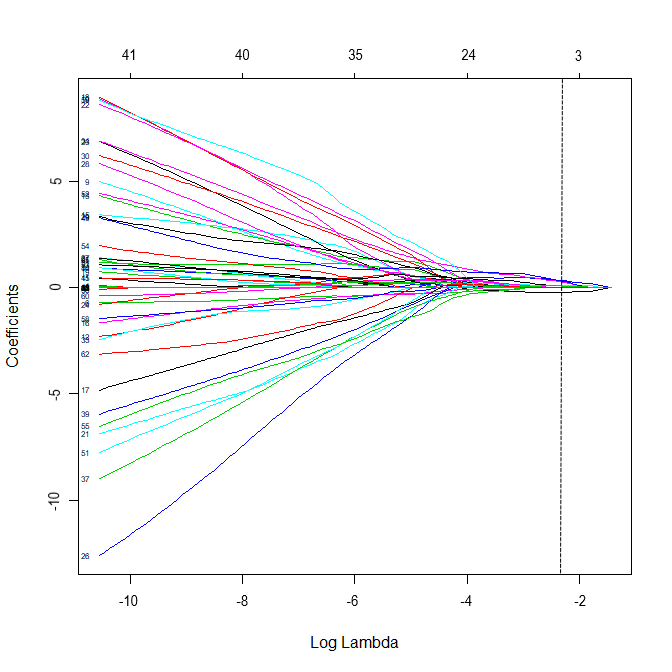


A B

**Supplementary Figure 2.** Feature distribution and selection based on the least absolute shrinkage and selection operator (LASSO) algorithm.

(A) To determine the best features combination for building the Radscore, the control parameter λ value in the LASSO model was selected via 10-fold cross validation with minimum criteria. The x-axis is the value of log (λ) and the y-axis is the binominal deviance in the 10-fold cross-validation. The upper x-axis is the number of non-zero-coefficient features with a given λ. The dotted line indicated the average binominal deviance value with the vertical bars showing the upper and lower boundaries. The left vertical dotted line defined the λ with the least binomial deviance. The right vertical dotted line indicates the largest value of λ such that the binominal deviance is within one standard error of the minimum binominal deviance and was set (log(λ min)=-2.46) in this study. (B**)** The LASSO coefficient profiles of the 704 radiomic features. The figure showed the feature coefficient change with the fine-tuning of λ value. The dotted line was plotted at the λ value determined in (A) resulting 5 non-zero-coefficient radiomic features.

1. **Correlation of five features**

The correlation coefficient between each pair is less than 0.75 (Figure S3). After calculating the collinearity of the five features through the VIF function in R, the values of the five features are 2.46, 1.46, 2.77, 1.80, and 2.31 respectively, which are all less than 10, that means there is no collinearity between the five features.


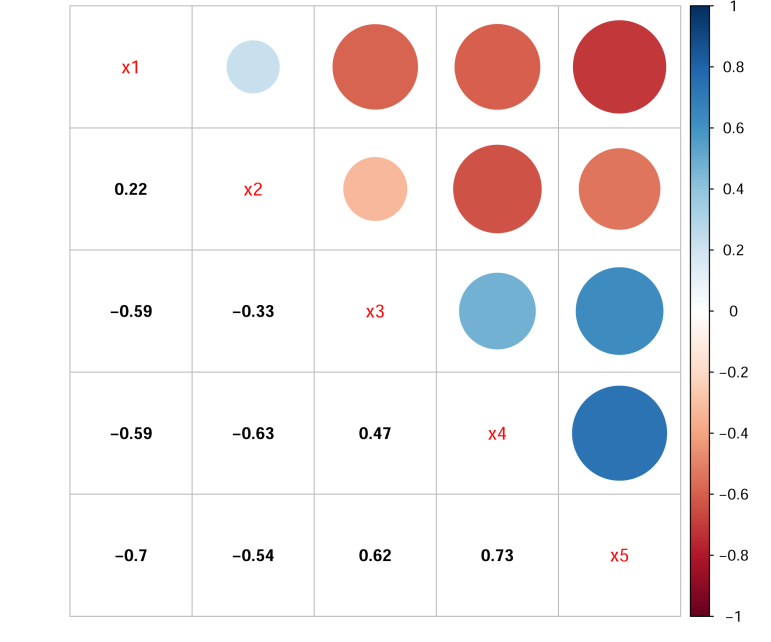


**Supplementary Figure 3.** Correlation of the features. The x1-5 represents the five features: Uniformity, SVR, CP_all_o1_SD, Correlation_a135_o1, and Inertia_a90_o4, respectively，the correlation coefficient between each pair is less than 0.75. There is no collinearity after verifying the correlation of the five features.

1. **Comparsion of Nomogram, Radscore and kinetic curve pattern**

The Delong’s test was performed to compare the AUCs of Nomogram, Radscore and kinetic curve pattern. The Nomogram had a significant improvement than the radscore and kinetic curve pattern alone in the training corhort (*p*<0.05). However, the improvement of the Nomogram model was not significant compared to radscore alone in the validation cohort (*p*=0.36). We speculate that this may be caused by the small sample size of the validation cohort (n=44). Detailed in Table S1 and S2.

Supplementary Table 1. Comparison of ROC curves in training cohort

| Nomogram ~ Radscore | |
| --- | --- |
| Difference between areas | 0.0447 |
| Standard Error ^a^ | 0.0220 |
| 95% Confidence Interval | 0.00168 to 0.0877 |
| z statistic | 2.037 |
| Significance level | P = 0.0417 |
| Nomogram ~ Kinetic_curve_pattern | |
| Difference between areas | 0.125 |
| Standard Error ^a^ | 0.0329 |
| 95% Confidence Interval | 0.0602 to 0.189 |
| z statistic | 3.786 |
| Significance level | P = 0.0002 |
| Radscore ~ Kinetic_curve_pattern | |
| Difference between areas | 0.0800 |
| Standard Error ^a^ | 0.0500 |
| 95% Confidence Interval | -0.0179 to 0.178 |
| z statistic | 1.601 |
| Significance level | P = 0.1093 |

Supplementary Table 2. Comparison of ROC curves in validation cohort

| Nnmogram ~ Radscore | |
| --- | --- |
| Difference between areas | 0.0434 |
| Standard Error ^a^ | 0.0470 |
| 95% Confidence Interval | -0.0487 to 0.136 |
| z statistic | 0.923 |
| Significance level | P = 0.3559 |
| Nnmogram ~ Kinetic_curve_pattern | |
| Difference between areas | 0.122 |
| Standard Error ^a^ | 0.0608 |
| 95% Confidence Interval | 0.00281 to 0.241 |
| z statistic | 2.006 |
| Significance level | P = 0.0448 |
| Radscore ~ Kinetic_curve_pattern | |
| Difference between areas | 0.0785 |
| Standard Error ^a^ | 0.0975 |
| 95% Confidence Interval | -0.113 to 0.270 |
| z statistic | 0.806 |
| Significance level | P = 0.4205 |
